# A Review of Temporary Cardiac Pacing Wires

**Published:** 2007-01-01

**Authors:** Peter McCann

**Affiliations:** North West Deanery, Manchester, UK

**Keywords:** pacing-wire, complications, vein, specialist, review

## Abstract

**Aims:**

This review aims to tabulate data from all available studies of temporary cardiac pacing wires. Particular aims were to determine the best route of venous access and find ways to reduce complications. The review set out to see if specialist doctors are better at inserting wires than non-specialist doctors. In addition, a contemporary study of wire insertion has been performed to compare modern practice in the UK with the previous studies.

**Methods:**

A literature search produced 15 studies available for inclusion. Over 3700 patients from 1973 to 2004 were included. The data was tabulated and attention was given to the route of venous access, the complication rates and whether a specialist or non-specialist doctor had inserted the wire.

**Results:**

Internal jugular veins are associated with lowest complication rates and ease of access. Antecubital fossa veins have the highest complication rates. Complication rates are high, especially infections and failure to secure access. Specialist doctors have lower rates of complications than non-specialist doctors. Elderly patient suffer the highest complication rate. Our study showed comparable results to the previous studies.

**Conclusion:**

Internal jugular veins are the preferred route for access followed by subclavian and femoral veins. The right side should be used when possible. The use of antibiotics and ultrasound probes must be contemplated for all wire insertions. Alternatives to wire insertion (especially in the elderly) must be seriously considered. Setting up an on-call rota would provide experienced doctors to reduce complication rates.

## Aims

Temporary cardiac pacing wires have existed for almost 50 years but their use remains controversial. Guidelines do exist for their use, but most of the recommendations come from clinical experience rather than scientific trials [1].

Over the past 30 years there have been several studies that look at various aspects of temporary pacing and this review incorporates 15 studies involving over 3700 patients. In addition, we have conducted a local study to compare our results with the other studies.

Particular aims of the review are to tabulate the available data in a simple format, so that all the studies may be reviewed easily.

It would be helpful to know which venous route is the best to secure access. Also, if the commonest complications can be identified, we may be able to take measures to minimize them. It would be useful to see if complications increase as the patients' ages increase because this could guide management of very elderly patients.

In the UK, most temporary cardiac pacing wires are inserted by generalists (that is general internal medicine physicians with little training inserting wires), whereas in mainland Europe and USA the trend has been towards the procedure being the sole domain of the specialist (cardiologist or anaesthetist). The review aims to determine if specialists should be inserting wires instead of generalists.

## Methods

Temporary cardiac pacing wires are usually inserted in an emergency situation. It is a procedure not practiced often, with the average general internal medical doctor in the UK performing less than five per year. Many of the studies therefore involve quite small numbers. The largest trial that exists in the literature is over 20 years old, although it has over 1000 patients. [2]

A literature review was performed. Medline, Embase, PubMed and the Cochrane library were all searched as well as the Internet using Google and Yahoo search engines. A variety of search words were used, with combinations of 'temporary, cardiac, pacing, wire(s), and pacemaker(s)'.

To minimize publication bias, unpublished data was searched for, especially as abstracts to conferences and meetings. Foreign language papers were also searched.

The main details from the papers were tabulated, starting with the earliest in 1973. The year and centre of the trial was recorded, as were the number of patients involved and the average age. It was often possible to determine which venous route was chosen for insertion of the wire. Many of the papers quoted a complication rate for the procedure.

A specialist study has been taken as one where the doctors inserting the cardiac wires were either cardiologists or anaesthetists. A generalist study is where the doctors have been physicians on a general internal medical take with no particular expertise in central line placement or temporary wire placement. A couple of studies had mixed sets of doctors inserting wires, so these have been grouped as 'mixed'.

In addition to the 15 review studies, the author of this review performed a retrospective audit of 56 consecutive patients from a modern UK hospital trust (East Lancashire NHS Trust) who underwent temporary cardiac pacing. The doctors inserting the wires were mostly specialist registrars or senior house officers with limited experience of inserting pacing wires.

Any patient that had a temporary cardiac pacing wire inserted at the trust in 2004 was included. Details of the procedures were taken from temporary pacing register and in the patient's case-notes. Ethical approval was not sought, as this was a retrospective audit with no direct influence on the patient's treatment.We do not usually seek ethical approval for retrospective reviews or retrospective audits as of the time of these studies.

There were no exclusion criteria. The route of venous access was recorded, as was the overall complication rate. Complications were defined as any adverse event that occurred because of the wire during the hospital stay. The results are included at the bottom of the first table.

Most of the data from the different studies could not be directly compared so that a meta-analysis was not possible. This is because none of the studies were randomised and different authors had different definitions of what a complication was.

## Results

Table 1 summarises the studies that have looked at temporary cardiac pacing wires.

### Route of Venous Access

The British Cardiac Society recommends the right internal jugular route as most suitable for the inexperienced operator because this offers the most direct route to the right ventricle, and is associated with the highest success rate and fewest complications [17]. As a result of five years of temporary pacing experience in a coronary care unit setting, Hynes also recommend the right internal jugular route (RIJ) [3].

Upon closer inspection of Hynes' paper very few patients had right internal jugular lines and the route was used increasingly throughout the five year period (i.e. in 1976, 252 lines were inserted, none via the RIJ approach. In 1980 only 162 lines were inserted, 54 via the RIJ). This might be because they became more selective with their patients or simply much better at putting in RIJ lines. However, the RIJ was associated with the lowest rate of loss of ventricular capture compared to other routes (7.2% loss at 3 days compared to average of 11.5%; antecubital loss was highest at 12.7%). RIJ lines had less than half the complication rate of antecubital fossa lines (8.1% versus 17.2%).

Ayerbe showed that for 99% of their 530 patients, the femoral route caused only 19% of complications [15]. The study is slightly ambiguous though because deaths are not included (taking the risks up to 25%). The authors' use of words such as 'serious' and 'severe' to describe complications could be seen to be ambiguous.

Since 1987, there have been no studies that have used the antecubital fossa veins for access. Subclavian vein access has always been popular. In the East Lancashire study most wires were inserted via the right internal jugular route, followed by the subclavian vein.

### Complications

Complications are common, occurring in 10% to 59.9% of procedures. The main problem with 'complications' is that every author uses different definitions. In some papers a complication may be minor: a localized infection or a friction rub. In other papers these minor complications are not recorded at all. If the pacemaker lead becomes dislodged at day 4, is this a complication or not? Some authors say yes, others no. This all leads to a great challenge when trying to compare papers.

The commonest complications were failure to secure venous access, failure to place the lead correctly, sepsis, puncture of arteries, lungs or myocardium and life-threatening arrhythmias (VF, VT). Table 2.

Most infections are caused by Staphylococcus epidermidis [18]. Complication rates from one third to over a half of all procedures were documented by Lumia [3], Austin [4], Winner [8], Volkmann [9] and Andrews [11]. Complication rates seem to be just as high now as they were 30 years ago. The complication rate in the East Lancashire study was 32%.

**Figure 1:**
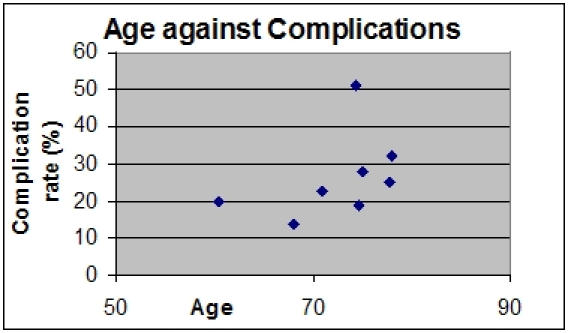


Figure 1 illustrates the fact that as patients get older, there is a trend towards complication rates increasing.

### Specialist versus Generalist

**Figure 2:**
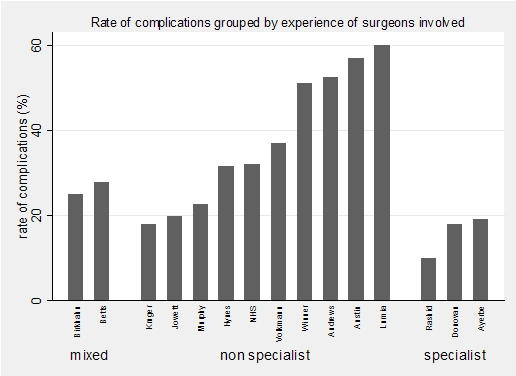
Complications of different operators

Figure 2 illustrates all of the studies in the review. It is clear from the table that specialists have the fewest complications, followed by both specialists and generalists combined, with generalists on their own experiencing highest complication rates. The East Lancashire study (labeled NHS below) is in the middle of the non-specialist bars.

A recent study in the UK at five hospitals in the Wessex Region showed that 144 procedures were performed on 111 patients (median age 75 years). Their complication rate was high at 32%; complications were significantly reduced if an experienced operator inserted the wire [14].

In a 2004 study, it was concluded that emergency trained physicians were proficient at temporary pacemaker insertion. From 117 wires the emergency trained physicians only inserted 30. They had 9/30 = 30% complications compared with 20/87 =  23% for the cardiologists [16].

### Indications for temporary wire insertion

The commonest indication for insertion of a temporary cardiac pacing wire is 3rd degree heart block. There are various other reasons why a wire was deemed to be necessary, such as failure of a permanent pacemaker, sick sinus syndrome and for sinus pauses. In the East Lancashire study wires were only inserted for third degree heart block or other bradycardias.

## Conclusions

Making any worthwhile conclusions from the different studies needs an appreciation that they have been performed over a 30 year period and that each study has looked at different aspects of the insertion of temporary cardiac pacing wires. In addition there have been advances in the treatment of cardiac patients including thrombolysis, increased use of anti-platelet agents, statins, ACE-inhibitors etc. over the past 30 years which is the main reason why less patients today have temporary cardiac pacing wires inserted. It is unlikely that a large study of over 1000 patients will ever be done again.

### Route of Venous Access

Most centres tended to favour one route of access over others. The papers demonstrate that antecubital fossa lines should not be inserted if possible because they have the highest complication rates. The right-sided veins are preferred over the left because permanent systems are usually inserted on the left side and because it is often technically easier from the right side.

There was quite a high failure rate in securing access in several of the studies (average 15%, range 6-40%). The use of ultrasound probes to help with securing venous access would be helpful (as recommended by NICE guidelines). They are however expensive items of equipment and some training in their use is recommended.

Balloon flotation devices have been suggested to help with positioning of the wire but that would mean having experience with yet another device and this is unlikely to be adopted. Our East Lancashire study shows that wires are being inserted in accordance to guidelines and also in line with the results of this review.

### Complications

The average complication rate is 26.5%. Because inserting temporary cardiac pacing wires is often an emergency procedure in elderly, frail patients it is difficult to know how many excess deaths are caused by inserting the wires themselves. In the East Lancashire study the complication rate was 32% but on the low end compared to other non-specialised centres.

Sepsis is a very common complication that can have serious repercussions such as removal of the wire, delay if a permanent system is needed, prolonged hospital stay and even death. Stringent aseptic techniques must be followed but there is no evidence that prophylactic antibiotics reduce the incidence of sepsis.

Complications arising from incorrect placement of the wire (failure of pacing, arrhythmias, perforations) will be reduced with increased training of the operators.

Murphy [19,20] stated that expecting trainees in medicine to perform transvenous pacing was no longer acceptable. He explained how in the USA and the most of Western Europe the procedure is the domain of the cardiologists. 22 years ago, Hynes concluded that training with regards to pacing wires was inadequate and needed addressing urgently [3].

In the USA it is recommended that to perform transvenous temporary pacing wires, the operator should be competent in placing intracardiac flotation catheters (a minimum of 25 procedures) [21]. Then a further 10 supervised pacing wires is needed to be deemed competent (in total 35 procedures) [22]. In addition the physician should be regularly assessed to ascertain continuing competency. The Council of the British Cardiac Society recommend that cardiology registrars perform a minimum of 25 temporary wires before being deemed competent. In 1995 the UK doctor had only seen two temporary pacing procedures and then performed two under supervision before doing them independently [19]. In the 10 years since then things have got worse because exposure is less [23].

This review can only endorse what previous authors have recommended. There is clearly resistance to changing the way temporary wires are inserted in the UK. It seems sensible that only trained, well-experienced operators should be inserting the wires.

### Specialists versus Generalists

The data seems to support the notion that specialists have a lower complication rate compared to generalists when inserting wires. This is not surprising because the specialist should have done many wires whereas sometimes the generalist might be a complete novice.

It is unrealistic to expect all general physicians (or specialist registrars) to be able to provide temporary wires and consultant cardiologist cover for most district general hospitals is not an option as there are not enough to go around. It would seem that if a wire must be inserted, advice from the tertiary centre should be sought. Before putting a wire in think:

1. Consider the need for a wire - is it absolutely necessary, can any possible alternative be used (see below)
2. Get the most experienced person available to put the wire in
3. Use the right internal jugular vein if possible (BCS recommendation)
4. Use an ultrasound machine if one is available
5. Be absolutely vigilant in aseptic technique

### Available alternatives

Transcutanous pacing has been less well studied in the literature although it has been shown to be both effective and relatively well tolerated. In a study of 21 patients it was demonstrated that the haemodynamic response to transcutaneous stimulation was similar to endocardial stimulation [24]. Transcutaneous pacing has advantages: its easy, quick, and safely applied. This makes it particularly valuable in emergency resuscitation especially if the healthcare professionals are less experienced. The study also showed that the use of transcutaneous pacemakers avoided the need for transvenous pacing wires altogether in 57 patients, from a total of 134 patients. Furthermore they found that temporary pacing was well tolerated in 73 out of 82 conscious patients.

Transoesophageal pacing (or Transoesophageal Atrial Pacing, TAP) is rarely used now. However, there is good evidence that it works well and is safe [25].  Unlike transvenous pacing, X-rays are not needed to check the position of the wire. Pacing can be achieved in 95% of patients. It produces a burning sensation in the chest that most patients can tolerate [26]. Unfortunately it is unlikely to become more widespread because of the lack of equipment and the lack of expertise in inserting such devices. In addition the studies that have looked into how effective it is have been mostly performed on anaesthetized patient and only in a prophylactic manner i.e. the wires were inserted in calm, controlled circumstances with co-operative patients. This does not mirror real life emergency procedures.

The patient who may benefit from temporary pacing can almost always rely on transcutaneous pacing for many hours successfully in the first instance; there is rarely need to rush in with transvenous pacing if the physician is not competent. Atropine can be used as a temporary measure to speed up some bradycardias.

The East Lancashire study states a complication rate of 32%. Although this is comparable with previous studies it still appears too high.

Temporary wire insertion will continue to be a challenge for the foreseeable future. If we are suggesting that only experienced operators are to insert wires an on-call rota system would have to be devised.  One could argue that they would not be needed very often so the call-out rate would be small. A group of ten experienced operators could cover a whole region reasonably.

On the other hand who would seriously give up extra time and commitment unless there was pecuniary recompense? The trusts would have to pay for the experienced operators. If only experienced operators are inserting wires, how would novice doctors ever get exposure to inserting wires?

## Figures and Tables

**Table 1:**
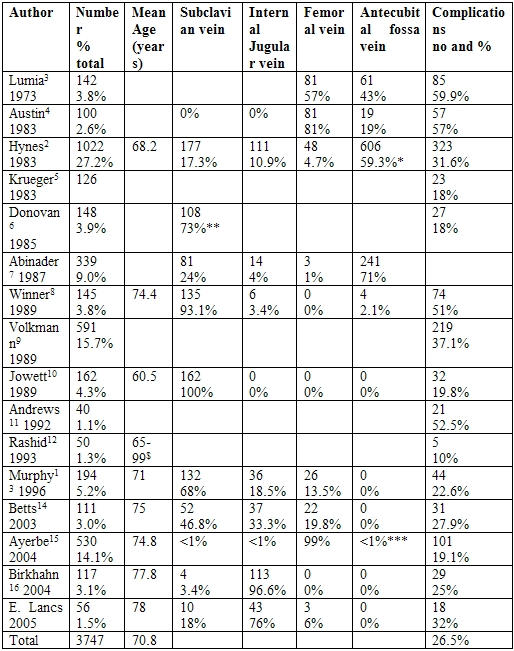
Study data tabulated

* only 942 of the 1022 patients had a venous route recorded ** again, not all the venous routes were identified *** exact numbers not stated $ mean age not stated

**Table 2:**
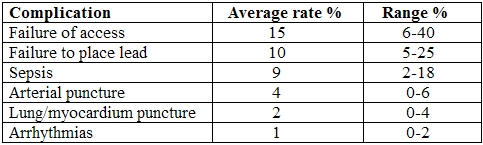
Commonest complications of temporary pacing wire insertion.
